# The Effect of *Plantago major* Hydroalcoholic Extract on the Healing of Diabetic Foot and Pressure Ulcers: A Randomized Open-Label Controlled Clinical Trial

**DOI:** 10.1177/15347346211070723

**Published:** 2022-01-19

**Authors:** Mustafa Ghanadian, Rasool Soltani, Alireza Homayouni, Farzin Khorvash, Soroush Mohammadi Jouabadi, Moein Abdollahzadeh

**Affiliations:** 1 Department of Pharmacognosy, School of Pharmacy, 48455 Isfahan University of Medical Sciences, Isfahan, Iran; 2 Department of Clinical Pharmacy and Pharmacy Practice, School of Pharmacy, 48455 Isfahan University of Medical Sciences, Isfahan, Iran; 3 Infectious Diseases and Tropical Medicine Research Center, Isfahan University of Medical Sciences, Isfahan, Iran; 4 Department of Pharmaceutics, School of Pharmacy, 48455 Isfahan University of Medical Sciences, Isfahan, Iran; 5 Research and Development Department, Goldaru Pharmaceutical Company, Isfahan, Iran; 6 48455 Nosocomial Infection Research Center, Isfahan University of Medical Sciences, Isfahan, Iran; 7 6993 Department of Epidemiology, Erasmus Medical Center, Rotterdam, the Netherlands; 8 Students Research Committee, School of Pharmacy, 48455 Isfahan University of Medical Sciences, Isfahan, Iran

**Keywords:** *plantago major*, healing, diabetic foot ulcer, pressure ulcer, clinical trial

## Abstract

**Aims:** Diabetic foot ulcer (DFU) and pressure ulcer (PU) both are common types of ulcers worldwide. The wound healing effect of *Plantago major* leaves has been shown in a few animal studies. This study aimed to evaluate the clinical efficacy of *P. major* hydroalcoholic extract on DFU and PU healing. **Methods:** In this clinical trial, patients with DFU or PU who met the inclusion criteria were randomly assigned to drug (*P. major*) or control groups. For patients in the drug group, Plantago extract 10% topical gel was applied on the wound once daily concurrent with dressing and routine wound care for two weeks, while for the control group, an appropriate novel dressing was used along with routine wound care for the same duration. The percentage of wound size reduction at the end of the seventh and 14th days of intervention was recorded and compared between the groups. **Results:** Fifty and 44 patients in drug and control groups, respectively, completed the interventions. Plantago extract gel significantly resulted in more reduction in the wound size compared to control at the end of the first (64.90 ± 29.75% vs. 33.11 ± 26.55%; *P* < 0.001) and second week (86.85 ± 24.34% vs. 52.87 ± 32.41%; *P* < 0.001). Furthermore, the number of patients with complete wound healing in the drug group (n = 32, 64%) was significantly more than the control group (n = 9, 20.45%; OR: 3.129, 95% CI: 1.685-5.809, *P* < 0.001). **Conclusion:** The use of 10% topical gel of *P. major* leaf extract results in the acceleration of DFU and PU healing. **Key points:** Application of *P. major* topical gel results in the acceleration of diabetic foot ulcer and pressure ulcer healing.

- *P. major* extract helps reducing the wound's erythema.
- *P. major* leaf extract assists decreasing the wound size.
- The number of patients completing wound healing process is higher among whom undergoing *P. major* dressing.

## Introduction

Wound healing and repair of damaged tissue are major problems.^1^ The global prevalence of diabetic foot ulcers (DFUs) is 6.3%.^2^ Over 15% of diabetic patients suffer from DFUs which are responsible for more than 80% of non-traumatic lower-extremity amputations. Peripheral neuropathy, peripheral arterial disease, and foot discomfort are considered some risk factor for this type of ulcer.^3^

Pressure ulcer (bedsore; PU) is also one of the common problems in healthcare centers. The reports show that the incidence of PU is about 0.4% – 38% in hospitals, 2.2% – 39% in long-term care facilities, and 0 to 17% in home.^4^ Several different factors contribute to the development and formation of bedsores, including pressure which is the most important external cause.^4^ In spite of implementing preventive measures for reduction of PU formation and efforts for its appropriate treatment, this type of ulcers remains common in the community and hospital converting to chronic wounds with challenging treatment and high rate of recurrence.^5^

Several plants have the potential for wound healing and have been used traditionally for this purpose.^6^ For example, the healing effect of banana leaf dressing on the partial thickness burn wounds has been shown.^7^ *Plantago major* L. is a plant from the Plantaginaceae family located in a vast region of Europe and Asia as well as North Africa and North America.^8^ Several experimental and animal studies have shown the beneficial effect of this plant on wound healing.^9–12^ Different mechanisms have been suggested for the wound healing effect of *P. major*, including antioxidant, anti-inflammatory, and antimicrobial effects due to its polyphenolic compounds,^12–14^ and induction of fibroblasts proliferation.^15^ However, there is no clinical study in this regard, Therefore, due to sufficient laboratory and animal evidence for *P. major* wound healing effect and lack of human studies in this case, and on the other hand, the high prevalence of DFUs and bedsores as well as presence of challenge in the treatment of these ulcers, the present study aimed to investigate the clinical effectiveness of the leaf extract of this plant on the healing of these two types of ulcers.

## Materials and Methods

### Extraction of the Plant Material

After collecting the plant in Kermanshah province, Iran, and its identification by the Pharmacognosy department of Pharmacy faculty at Isfahan University of Medical Sciences (IUMS), the leaves were crushed by a hammer mill. The extraction was performed by the maceration method, using ethanol 70% (Dr Mojallali Co., Iran). Then the extract was concentrated using a rotary evaporator and kept in the refrigerator.

### Extract Standardization

Five mL of the extract (equivalent to 0.5 g of the powdered extract) was dissolved in 10 mL of 96% ethanol (Dr Mojallali Co., Iran) in a 100-mL volumetric flask. The distilled water was gradually added to it up to a total volume of 100 mL. Within each of the three test tubes, 20 μL of the obtained sample and 1.58 mL of water were added. In the next step, after adding 100 μL of Folin-Ciocalteu reagent and 0.3 mL of sodium carbonate solution to each tube, they were stirred well and placed in the closet (preferably dark environment) for 2 h. Following the preparation of five different concentrations of Gallic acid, reading their absorbance in 765 nm, and plotting the corresponding standard absorbance-concentration curve, the absorbance of samples in 765 nm was read against the blank (water) and the extract total phenolic content was determined.

### Preparation of the Topical gel

At first, a certain amount of hydroxypropyl methylcellulose (HPMC), methyl paraben, and propyl paraben were added to about two-thirds of the extract at 70 °C. Then, it was stirred well to wet the HPMC particles. The remaining amount of the cooled extract was then added and mixed well until a uniform gel formed. Of note, the water content of the extract was considered as the required water for the formulation. The concentrations of ingredients (%v/v) at the final formulation were: HPMC 6%, propyl paraben 0.02%, methyl paraben 0.18%, the herbal extract 10%. Of note, the obtained gel was kept in the refrigerator overnight.

### Study Design

This study was a randomized open-label controlled clinical trial conducted from May 2020 to March 2021 at Al-Zahra Hospital of Isfahan (affiliated to IUMS). The study was registered in the Iranian Registry of Clinical Trials with registration ID IRCT20150721023282N12. Also, the ethical approval was received from the ethics committee of IUMS (ethics reference number: IR.MUI.RESEARCH.REC.1399.146). Informed written consent was obtained from all participants.

### Patients

The patients were selected from those hospitalized in different wards of the hospital. The demographic and clinical characteristics of the subjects including age, sex, and type of ulcer were recorded.

The following criteria were considered for patients to be included in the study: 1) having grade 1 or 2 diabetic foot ulcer based on the Wagner classification system,^16^ or stage 2 or 3 pressure ulcer based on NPIAP (National Pressure Injury Advisory Panel) staging system,^17^ in one or more areas; 2) ankle-brachial index (ABI) > 0.9; 3) not smoking at the moment; 4) not using anticoagulant drug within the last three months; 5) not using immunosuppressive drug within the last 3 months; 6) lack of coagulation disorder (e.g., hemophilia); and 7) no use of any drug affecting wound healing (phenytoin, glucocorticoids, anticancer drugs, and NSAIDs) in the last 3 months.

Patients who showed any allergic reaction to the herbal topical gel, those who required urgent re-vascularization due to severe limb ischemia, and those who were discharged from the hospital before the end of intervention, were excluded from the study.

### Interventions and Assessments

The goals and method of conducting the trial were fully explained for the patients before their inclusion to the study. Patients who entered the research, were randomly assigned to two groups of drug (Plantago) and control. Simple randomization method was used for random allocation, so that the patients with even and odd medical profile number were assigned to drug and control groups, respectively.

For people in the drug group, a 10% Plantago extract gel was applied on the wound surface once a day after saline irrigation and cleansing, followed by dressing with sterile gauze and bandage. Surgical wound debridement and antibiotic therapy were performed as necessary. For patients of control group, an appropriate novel wound dressing (selected by the hospital wound care team according to the wound characteristics, including alginates or foams for moderate to high exuding wounds; honey for sloughy, low to moderate exuding wounds; hydrocolloids for clean, low to moderate exuding wounds; and hydrogels for dry, low to moderate exuding wounds; with silver-containing form of each dressing being used for infectious wounds) was applied once daily along with all common wound care measures including saline irrigation and cleansing as well as surgical wound debridement and antibiotic therapy as necessary. The interventions were applied for two weeks in both groups.

Before the start of the mentioned interventions, the wound size was determined by multiplying the largest length and maximum wound width per cm, and also, the presence or absence of erythema (redness) around the wound was determined and recorded. At the end of days 7 and 14 (end of the intervention), these indicators were re-determined and documented. The percent reduction of wound size was calculated using the following formula:

Percent reduction in size = [(primary size – final size)/primary size] × 100

Also, the number of cases of complete wound healing during the intervention was recorded in both groups.

The primary outcome measure was the mean percent of change in the wound size at the end of the first and second weeks, while the number of cases of erythema around the wound at the end of the first and second weeks and the number of cases of complete wound healing over the study period were considered as the secondary outcome measures. A per-protocol analysis was performed to compare the outcome variables between the groups.

### Statistical Analysis

Statistical analysis was performed using SPSS software version 24. The Smirnov-Kolmogorov test was used to determine the distribution pattern of quantitative data (normal vs. non-normal).

To compare the mean percent of change of wound size between the two groups at the end of the first week, due to the normal distribution of data, the Independent-Samples T-Test was used, whereas at the end of the second week, due to non-normal data distribution, the Mann-Whitney U Test was performed for comparison. Fischer's exact test was utilized to compare the number of erythema cases as well as the number of complete wound healing cases between the two groups.

*P*–values less than 0.05 were considered as significant

## Results

### Extraction and Standardization

After the Plantago extraction procedures, 7.5 L of condensate extract was obtained. Assessing the total phenolic content according to the described method, it was determined that each 5 mL of the extract contained 0.019 mg of polyphenolic compounds.

### Patients

**Figure 1.**
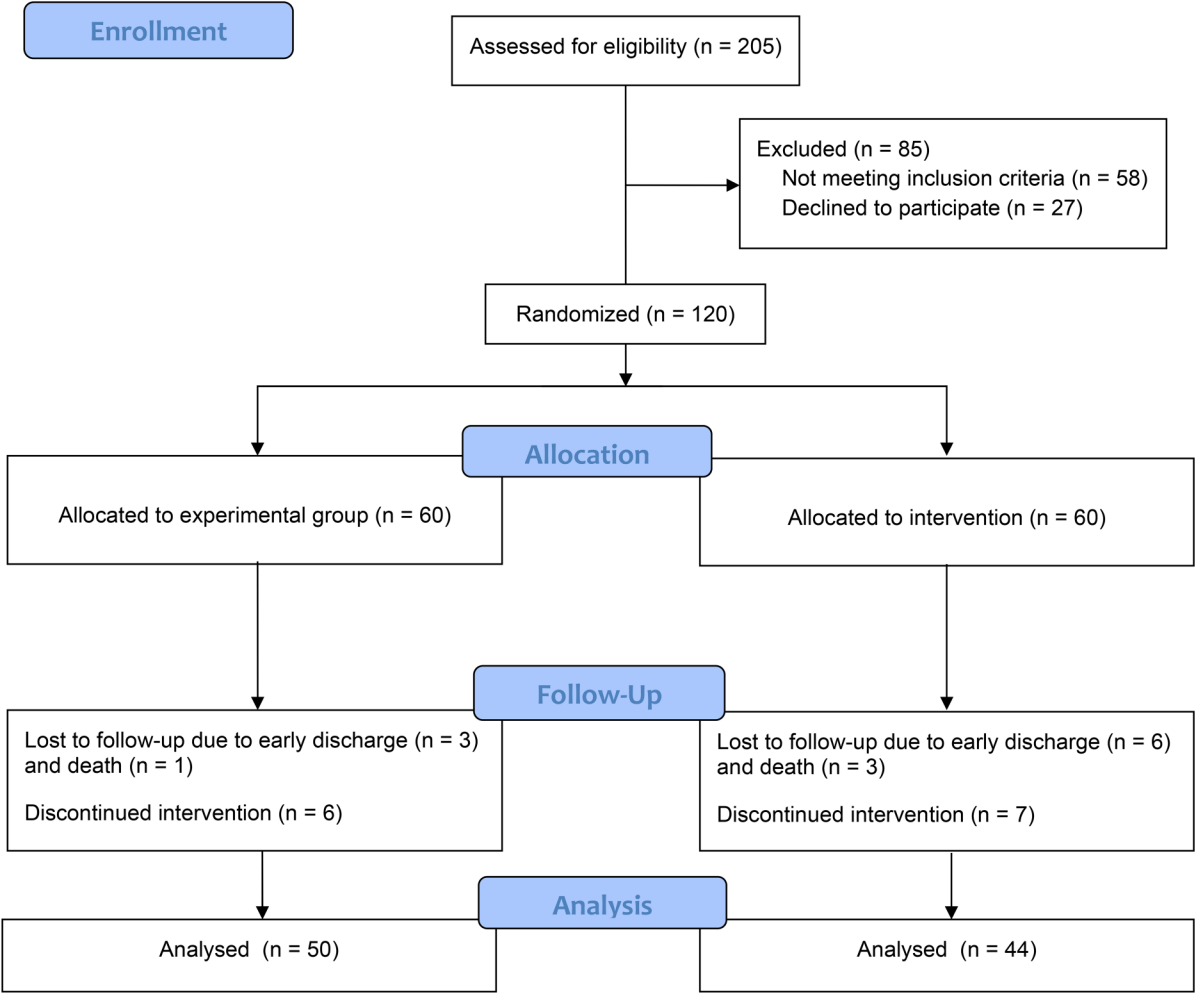
Consort flow diagram.

During the study, a total of 205 patients were evaluated, of whom 147 patients met the inclusion criteria. A total of 120 patients were randomly assigned into two groups of drug and control. Ten and 16 patients from drug and control groups, respectively, were dropped out due to either lack of cooperation, discharge from the hospital before the trial completion, or death. So, 50 and 44 patients in drug and control groups, respectively, completed the interventions (Figure 1).

As shown in the Table 1, there was no significant difference in the baseline demographic and clinical characteristics of study patients.

**Table 1.** Baseline Demographic and Clinical Characteristics of Study Patients.

Parameter	Drug group (n = 50)	Control group (n = 44)	p-value
Sex (n)			
Male	29 (58%)	30 (68%)	0.393
Female	21 (44%)	14 (32%)	
Age (years; mean ± SD)	56.82 ± 17.37	62.31 ± 9.51	0.057
Type and grade of the ulcer (n)			
DFU grade 1	1 (2%)	0	
DFU grade 2	15 (30%)	12 (27%)	0.169
PU stage 2	4 (8%)	0	
PU stage 3	30 (60%)	32 (73%)	
Wound size (cm^2^)	10.16 ± 9.40	9.57 ± 9.45	0.122
Erythema around the wound (n)	19 (38%)	26 (59%)	0.062

DFU, diabetic foot ulcer; PU, pressure ulcer.

### Outcome Measures

**Figure 2.**
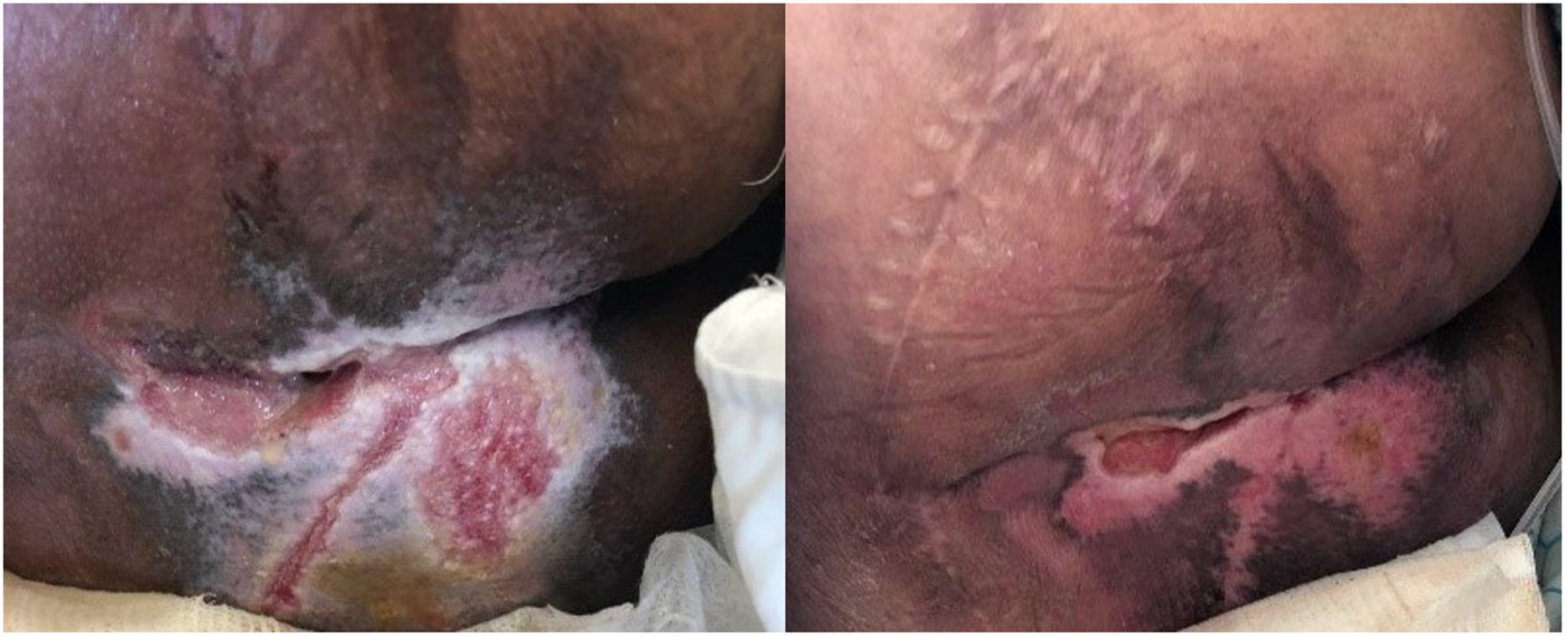
The effect of Plantago extract gel on reduction of pressure ulcer size: before application (left), after two weeks of application (right).

Table 2, shows the obtained results of evaluated parameters at different time points over the study period and their comparison between the groups. It is discernible that, at the end of both the first and second weeks of intervention, the wound size reduced in both groups, with the percent of reduction being more at the second week. However, there was significantly more reduction of the wound size in drug group compared to the control at both time points (Figure 2). Furthermore, although there was no significant difference between the groups regarding the number of erythema cases at the beginning of the study (baseline, *P* = 0.062), at the end of the first and second weeks of the trial, it was significantly lower in drug group compared to the control group. Moreover, at the end of study, there were significantly more patients with complete wound healing in drug group (64%) compared with the control (20.5%).

**Table 2.** The Obtained Results of the Evaluated Variables and Their Comparison Between the Groups.

Parameter	Time	Drug group (n = 50)	Control group (n = 44)	*P-value*
Wound size reduction (%, mean ± SD)	first week	64.90 ± 29.75	33.11 ± 26.55	<0.001
	second week	86.85 ± 24.34	52.87 ± 32.41	<0.001
Erythema around the wound (n)	first week	12 (24%)	26 (59%)	<0.001 OR: 0.406 (95% CI: 0.234-0.705)
	second week	10 (20%)	23 (52%)	<0.001 OR: 0.383 (95% CI: 0.205-0.713)
Complete wound healing (n)	second week	32 (64%)	9 (20.50%)	<0.001 OR: 3.129 (95% CI: 1.685-5.809)

n, number of cases; OR, Odds Ratio; CI, confidence interval.

## Discussion

In this study, patients who used Plantago gel, compared to those who used novel wound dressing, had more percentage of wound size reduction as well as erythema decline by the end of the first and second weeks of treatment. Moreover, Plantago gel resulted in more cases of complete wound healing. These results indicate that Plantago leaf extract has a significant effect on the repair of DFUs and PUs.

To the best of our knowledge, this is the first clinical study evaluating the effect of *P. major* on the ulcer healing. However, some animal studies have been performed in this regard. In the study of Ghasemiboroon et al,^13^ the effect of the combination of cabbage, pomegranate, and Plantago on the wound healing was evaluated in rats. Based on the results, the percentage of wound healing (reduction in the wound size) in the third, sixth, ninth and 14th of the study was significantly more than the control group (pethadine), and on days 3, 6 and 9, it was more than the control groups.^13^ In another animal study by Mahmood and Phipps,^10^ the use of 5% and 10% Plantago leaf extract ointment accelerated the wound healing in rats compared to the control group (Vehicle alone).

In an investigation performed by Amini et al,^11^ although application of 20% and 50% Plantago solution on grade 3 burn wounds in rats for 3 weeks had no significant effect on the wound size in comparison to control groups (silver sulfadiazine ointment and eucerin), the pathological examination revealed beneficial effects in the wounds, including increased re-epithelialization and tissue granulation as well as increase in the number of capillaries in the wound site. Additionally, the in-vitro study of Zubair et al,^12^ showed positive effect of both water- and ethanol-based extracts of *P. major* leaves on the proliferation/migration of the oral epithelial cells, with the ethanolic extract having the most beneficial effects.

Therefore, our study confirms the results of most animal studies indicating significant effectiveness of *P. major* leaf extract in accelerating the healing of ulcers.

As mentioned previously, our research is the first human (clinical) study in this field. Of note, there is a clinical trial on the effect of Plantago extract mouthwash on chemotherapy-induced mucositis in cancer patients compared with sodium bicarbonate and chlorhexidine solutions.^18^ The results showed the positive effects of the extract in the acceleration of mucositis recovery and improvement of the related pain comparable to the two other solutions.^14^ These results are consistent with our outcomes indicating the healing potential of *P. major* leaf extract on wound and inflammation.

Different mechanisms have been suggested for the wound healing effect of *P. major*. In some studies, this effect has been attributed to polyphenolic compounds and antioxidant properties of the extract.^13^ Supporting this proposed mechanism, a study by Qin et al,^19^ showed the effects of green tea polyphenols on the wound healing in vitro and this effect was attributed to the antioxidant properties of these compounds. Indeed, oxidative stress and inflammation have been implicated in the wound healing process.^20^ Therefore, the antioxidant and anti-inflammatory agents could have a role in the acceleration of ulcer healing. A polyphenol compound in Plantago extract, called plantamajoside, has been introduced as an anti-inflammatory, anti-oxidant, and antibacterial substance and may be responsible for healing properties of the extract.^12,14^ It is also noted that the proteins extracted from Plantago extract affect the proliferation of fibroblasts resulting in the wound healing acceleration.^15^

Besides, Plantago extract has other effects, such as increased nitric oxide production (NO), which may play a role in the wound healing.^21^ Plantago flavonoids, including hispidulin and baicalein, have anti-inflammatory and anti-fungal properties. They also inhibit the release of prostaglandins and histamine, blocking their destructive effects on tissue.^22^ Furthermore, astringency effect of flavonoids,^23^ may play a key role in accelerating wound healing.

Predominantly, due to the high prevalence of diabetic foot and bedsore ulcers and the lack of appropriate response to current treatments together with the high cost of new dressings used in the treatment of such ulcers and according to the results of the prior reports and current study, it seems that the use of *P. major* extract topical products can be considered as a potential developing treatment. Further studies with larger sample size and longer duration are required to confirm this effect and to determine the ideal preparation and concentration as well as the optimum duration of its use for the best efficacy.

The main limitations of our study were relatively low sample size, lack of placebo and blinding (due to technical problems), short duration of intervention, and use of different types of novel dressing in the control groups (because of different characteristics of ulcers necessitating this). However, this is the first pilot clinical study showing the beneficial effects of *P. major* leaf extract on the healing of DFU and PU.

## Conclusion

The use of 10% topical gel of *P. major* leaf hydroalcoholic extract results in the acceleration of DFU and PU healing.

Trial Registration: The study was registered in the Iranian Registry of Clinical Trials with registration ID IRCT20150721023282N12 (https://www.irct.ir/trial/38487).

## Supplemental Material

sj-doc-1-ijlew-10.1177_15347346211070723 - Supplemental material for The Effect of *Plantago major* Hydroalcoholic Extract on the Healing of Diabetic Foot and Pressure Ulcers: A Randomized Open-Label Controlled Clinical Trial Supplemental material, sj-doc-1-ijlew-10.1177_15347346211070723 for The Effect of *Plantago major* Hydroalcoholic Extract on the Healing of Diabetic Foot and Pressure Ulcers: A Randomized Open-Label Controlled Clinical Trial by Mustafa Ghanadian, Rasool Soltani, Alireza Homayouni, Farzin Khorvash, Soroush Mohammadi Jouabadi and Moein Abdollahzadeh in The International Journal of Lower Extremity Wounds
